# Spatiotemporal disarray of inflammatory microenvironment following spinal cord injury

**DOI:** 10.4103/NRR.NRR-D-25-00822

**Published:** 2025-12-30

**Authors:** Jiawei Di, Yubao Lu, Senyu Yao, Haojie Zhang, Zhenming Tian, Longyou Xiao, Zhizhong Shang, Lei He, Mao Pang, Yang Yang, Liangming Zhang, Liumin He, Bin Liu, Limin Rong

**Affiliations:** 1 Department of Spine Surgery, The Third Affiliated Hospital of Sun Yat-sen University, Guangzhou, Guangdong Province, China; 2 Guangdong Provincial Center for Engineering and Technology Research of Minimally Invasive Spine Surgery, Guangzhou, Guangdong Province, China

**Keywords:** bioengineering, central nervous system, gene expression profiling, mesenchymal stem cells, neuroinflammatory diseases, neurosciences, organoids, precision medicine, regeneration

## Abstract

Spinal cord injury is a severe neurological condition with far-reaching consequences for both individuals and society. Its progression involves a complex interplay between the initial mechanical insult and inflammation-driven secondary injury. This review interprets recent studies on the spatiotemporal map of the inflammatory microenvironment in spinal cord injury and other neurological disorders, focusing on the latest mechanistic research and treatment options. The postinjury inflammatory microenvironment comprises diverse immune and glial cell populations whose phenotypes and functions change over time. The current consensus suggests that inflammation has a paradoxical nature: while it can limit lesion spread in the acute stage, chronic or dysregulated responses contribute to further neural damage through pathways such as excitotoxicity, oxidative stress, and glial scar formation. Methods such as spatiotemporal transcriptomic analyses and organoid-based models have improved our ability to resolve cell–cell interactions and discover new molecular targets. Numerous therapies targeting this microenvironment have been developed. Stem cell–based approaches, especially human umbilical cord mesenchymal stem cells, show promise in promoting tissue regeneration and immune regulation in experimental and early clinical studies. Bioengineering methods such as using biomaterial scaffolds and controlled drug release are being investigated to improve drug delivery and the injury microenvironment. Pharmacological efforts to modify cytokine networks, oxidative pathways, or immune checkpoints have achieved some success, hampered by patient heterogeneity and injury patterns. Persistent challenges include determining the context-specific mechanisms, identifying the best treatment windows, and combining a few methods for the best effect. In summary, detailed understanding of the spatiotemporal dynamics of post–spinal cord injury inflammation is essential for designing targeted interventions. Future directions include integrating multiomics datasets, identifying predictive biomarkers for patient stratification, and developing adaptive treatment protocols that fine-tune rather than suppress immune responses to promote neural repair and functional recovery.

**Facts**

• Recent research has clarified that the inflammatory response after spinal cord injury has spatiotemporal specificity; early inflammation can limit the spread of injury, but excessive or prolonged responses can exacerbate tissue damage and inhibit axon regeneration.

• Studies targeting the inflammatory microenvironment at different stages have revealed the dynamic roles of immune cells, such as microglia and macrophages, and their dual impact on nerve regeneration.

• Novel therapeutic strategies based on the spatiotemporal regulation of inflammation are focusing on stage-specific interventions, aiming to promote nerve repair and functional recovery through precise modulation of immune responses.

**Open questions**

• Which spatiotemporal biomarkers are most effective in accurately defining the timing and regions of inflammatory regulation?

• How can we optimize spatial transcriptomics and artificial intelligence-driven modeling techniques to predict the “optimal time window” for individualized interventions, while also considering immunological heterogeneity among patients?

• Which combination therapy regimens are best suited for specific stages and severities of spinal cord injury?

## Introduction

Spinal cord injury (SCI) is one of the most severely disabling neurological disorders, causing profound impairments in long-term quality of life (Lu et al., 2024, 2025b). This significant global burden poses a major challenge for healthcare systems and highlights the need for comprehensive rehabilitation and long-term care. The pathological course of SCI involves more than just the initial traumatic impact; it also includes secondary injuries resulting from inflammation, which play important roles in influencing the likelihood of neuronal growth and regeneration (Bradbury and Burnside, 2019). Inflammation following SCI is inherently dual-faceted: it may help prevent further lesion growth early after the injury, but excessive or prolonged inflammation can exacerbate tissue damage and slow new axon growth, partly by promoting glial scar formation (Yao et al., 2021; Tan et al., 2024). Understanding how the inflammatory environment changes over time and space is essential to comprehend the processes underlying neural damage and to guide the design of regenerative interventions based on this research.

Recently, the inflammatory microenvironment following SCI has been recognized as consisting of two components: activated resident immune cells and new, circulating immune cells entering the site of injury. Cytokines and chemokines work together in these processes, which collectively impede axon growth. Early treatment with anti-inflammatory agents may be beneficial and can prevent acute tissue damage; however, inflammation has occasionally produced unsatisfactory outcomes, which are associated with inadequate consideration of the timing and variability of inflammatory reactions. This indicates a gap in understanding how inflammation is spatiotemporally regulated (Benowitz et al., 2023). In accordance with the “spatiotemporal dislocation” of the inflammatory microenvironment, this article reviews the different cellular responses at each stage after injury and evaluates the beneficial and detrimental effects of each cell type on neuronal repair. The goals are to provide a comprehensive understanding of this dysregulation in an interactive manner, examine how these processes relate to regeneration, develop therapeutic methods designed to control inflammation with stage-specific, site-specific approaches, and connect all known molecular relationships to treatments for SCI in the clinical field.

## Search Strategy

A literature search was conducted in PubMed and Web of Science for studies related to the spatiotemporal disruption of the inflammatory microenvironment (STDIM) following SCI, covering the period from the inception of the databases to June 2025. The search terms included “SCI,” “inflammation,” “microenvironment,” and related synonyms. We excluded studies unrelated to the inflammatory microenvironment following SCI by reviewing titles and abstracts, and non-English publications were also excluded. However, studies on stem cell therapies for SCI, biomaterials, microenvironmental engineering treatments, and pharmacological approaches were included to explore strategies for repairing the inflammatory microenvironment. In total, 192 studies were included in this review.

## Spatiotemporal Organization of the Inflammatory Microenvironment in the Central Nervous System and Systemic Pathologies

### Structural components and pathophysiological evolution of the inflammatory microenvironment

Inflammation is a highly conserved biological response that has persisted throughout evolution, reflecting its fundamental role in defending against infection and supporting tissue repair. Across species, the core mechanisms of inflammation are preserved due to their essential contributions to host survival (Burini et al., 2020; Medzhitov, 2021). This multifaceted response involves the activation of both immune and non-immune cell populations, which work together to eradicate pathogens and facilitate tissue restoration, thereby protecting the organism from bacterial, viral, toxic, and other harmful insults (Botella Lucena and Heneka, 2024).

Following traumatic events in the central nervous system, such as SCI, this conserved response is similarly activated. After injury, there is a significant influx of cells, including microglia, macrophages, astrocytes, and neutrophils, as well as mediators such as cytokines, excitatory neurotransmitters, reactive oxygen species, and nitrogenous compounds. These elements collectively constitute the inflammatory microenvironment, influencing local conditions after injury through intricate interactions (Freyermuth-Trujillo et al., 2022). The resulting microenvironment is neither inherently harmful nor maladaptive; rather, it represents a survival response aimed at regulating cellular states, clearing damaged tissue, and promoting repair (Medzhitov and Iwasaki, 2024). The mediators and cell types within this microenvironment collaboratively participate in and impact the processes and outcomes of inflammation.

#### Spectrum of inflammatory mediators and cellular effectors cytokine networks

Cytokines are soluble proteins and glycoproteins released by various cells and serve as signaling molecules mediating intercellular communication and regulating immune responses. In SCI, proinflammatory cytokines and chemokines directly influence the recruitment and activation of inflammatory cells, thereby affecting repair processes following injury (Becher et al., 2024). The patterns of cytokine and chemokine upregulation in serum and cerebrospinal fluid after SCI are analogous to those in the damaged tissue itself (Sterner and Sterner, 2022). Expression of the proinflammatory cytokine interleukin-1β (IL-1β) increases immediately following SCI, whereas mRNA expression of interleukin-6 (IL-6), another inflammatory mediator, peaks nearly 50-fold within 1 hour. Additionally, expression of tumor necrosis factor-alpha (TNF-α), a member of the death receptor TNF superfamily, also rises immediately after SCI. Immunoreactivity of proinflammatory cytokines (IL-1β, TNF-α, and IL-6) decreases within 24 hours and disappears by the second day (Saghazadeh and Rezaei, 2017). Generally, SCI severity closely correlates with the intensity of the acute inflammatory response. In individuals with severe paraplegia, CD68^+^ bone marrow-derived macrophages coexpress IL-1β and TNF-α, whereas moderate spinal ischemia‒reperfusion injury does not show this pattern (Zhang et al., 2025b).

Chemokines primarily facilitate cell migration by guiding recruited cells toward the source of increasing chemokine concentrations. The expression of CXCL-1, secreted by activated T cells to recruit monocytes, natural killer (NK) cells, and dendritic cells, rapidly rises and continues to increase within 3 to 6 hours after injury (Hassanshahi et al., 2013). Microglia and astrocytes release CCL2, which binds to CCR2 and promotes a substantial influx of monocytes to the inflammatory site (Rong et al., 2021; He et al., 2024). Furthermore, these cells produce CXCL8 (interleukin-8 [IL-8]), the key chemokine for neutrophil recruitment, which primarily induces neutrophil migration and degranulation via CXCR1 and CXCR2 (Dhayni et al., 2022).

(1) Excitatory neurotransmitter release and signaling

Excitotoxicity is a significant event mediated by inflammation following SCI that is primarily induced by the rapid release of extracellular glutamate. This process leads to the excessive activation of glutamate receptors, causing a swift influx of sodium (Na^+^) and calcium (Ca^2+^) ions into cells that results in ionic imbalances and cell death (Harris et al., 2025). In the central nervous system (CNS), glutamate is the most important excitatory transmitter and is important for signaling. A rapid increase in the glutamate concentration occurs specifically within the first few hours after SCI, and glutamate can also cause the death of motor neurons. Preclinical studies showed that reducing glutamate excitotoxicity 1 hour after SCI can help improve various healing parameters (Lima et al., 2021; Cao et al., 2024). The proinflammatory cascade seems to connect with the glutamate excitotoxicity caused by TNF-α, exacerbating secondary injury below the level of the lesion, but some projections of glutamatergic networks in the spine neurons are still able to transmit cortical commands from the forelimbs to the hindlimbs in rats (Hachem et al., 2023; Witkin et al., 2024). Therefore, restoring excitatory glutamatergic signals in the damaged spinal cord is possibly a strategy to restore function in these patients. Moreover, the activation of purinergic signaling is also needed after SCI. Adenosine triphosphate (ATP) is a neurotransmitter that has many regulatory effects on the CNS. One of these effects is the regulation of the variability of signal transduction between glial cells and neurons. The activation of P2X4 purinergic receptors can induce microglial activation and exacerbate apoptosis and neuronal loss (Cheng et al., 2023). After apoptosis occurs, damaged cells release more ATP, which induces nearby cells to release additional ATP. Furthermore, damaged cells release nucleotides into the surrounding area, encouraging even more ATP release from adjacent cells. Therefore, just hours after injury, the amount of extracellular ATP within and adjacent to the lesion continues to increase, affecting Na^+^, K^+^, and calcium (Ca^2+^) channels (Stefanova and Scott, 2022).

(2) Reactive oxygen and nitrogen species

Reactive oxygen species (ROS) and reactive nitrogen species (RNS) are generated early in the injury process, with oxidative and nitrosative stress resulting from an imbalance between the elevated levels of ROS and RNS and antioxidant defense mechanisms. Oxidative stress can be considered a double-edged sword (Murphy et al., 2022). In the early stages, low levels of oxidative stress may protect damaged tissues and cells; however, persistently high levels can exacerbate inflammation and cell death. Following SCI, secondary pathological changes such as hypoxia and hemorrhage lead to mitochondrial dysfunction and increased activity of ROS-generating enzymes, resulting in the excessive production of ROS. This overabundance of ROS induces oxidative stress and cell death (Yao et al., 2023; Yin et al., 2024). The excessive activation of inducible nitric oxide synthase (iNOS) generates large amounts of nitric oxide, which reacts with superoxide anions to form peroxynitrite, further promoting the peroxidation of DNA and lipids. The decomposition of oxygen radicals and peroxynitrite generates additional harmful free radicals, resulting in oxidative damage to cellular components (Hall, 2011; Yao et al., 2024).

(3) Immune and glial cell populations

The central canal is present in the center of the healthy spinal cord and is covered by ependymal cells (Rodrigo Albors et al., 2023). Gray matter surrounds the canal and contains many more neurons in the form of large cell bodies and dendrites in an H formation. The white matter composed of mostly tightly packed myelinated axons surrounds the gray matter (Hashmi et al., 2022). Oligodendrocytes cover larger axons with diameters greater than 0.2 μm in myelin (Basu et al., 2023), and these cells mostly reside in the white matter (Kuhn et al., 2019). Microglia and astrocytes are present in both gray matter and white matter and are involved in immune surveillance and metabolism (Brockie et al., 2021). The spinal cord has an extensive vascular network with fibroblasts and macrophages distributed throughout the interstitial tissue within the perivascular spaces surrounding the microvasculature (Dorrier et al., 2022).

When SCI occurs, damage arises from two main sources: primary injury and secondary injury. Primary injury typically results from compression–contusion forces, where displaced vertebral elements, including discs and ligaments, directly compress the spinal cord, causing immediate harm with or without sustained pressure (Alizadeh et al., 2019; Guest et al., 2022). The mechanisms include shearing, tearing, acute stretching, and rapid acceleration–deceleration, which seldom result in complete anatomical transection but frequently result in axonal disruption, microvascular rupture, and necrosis (Awad et al., 2020). Because of the destructive potential of these mechanisms, early surgical decompression—ideally within 24 hours—is widely considered the most effective intervention to limit further damage. The extent of the primary injury remains a strong prognostic factor, as greater trauma generally correlates with more severe neurological deficits (Ahuja et al., 2017; Badhiwala et al., 2021).

**Figure 1:**
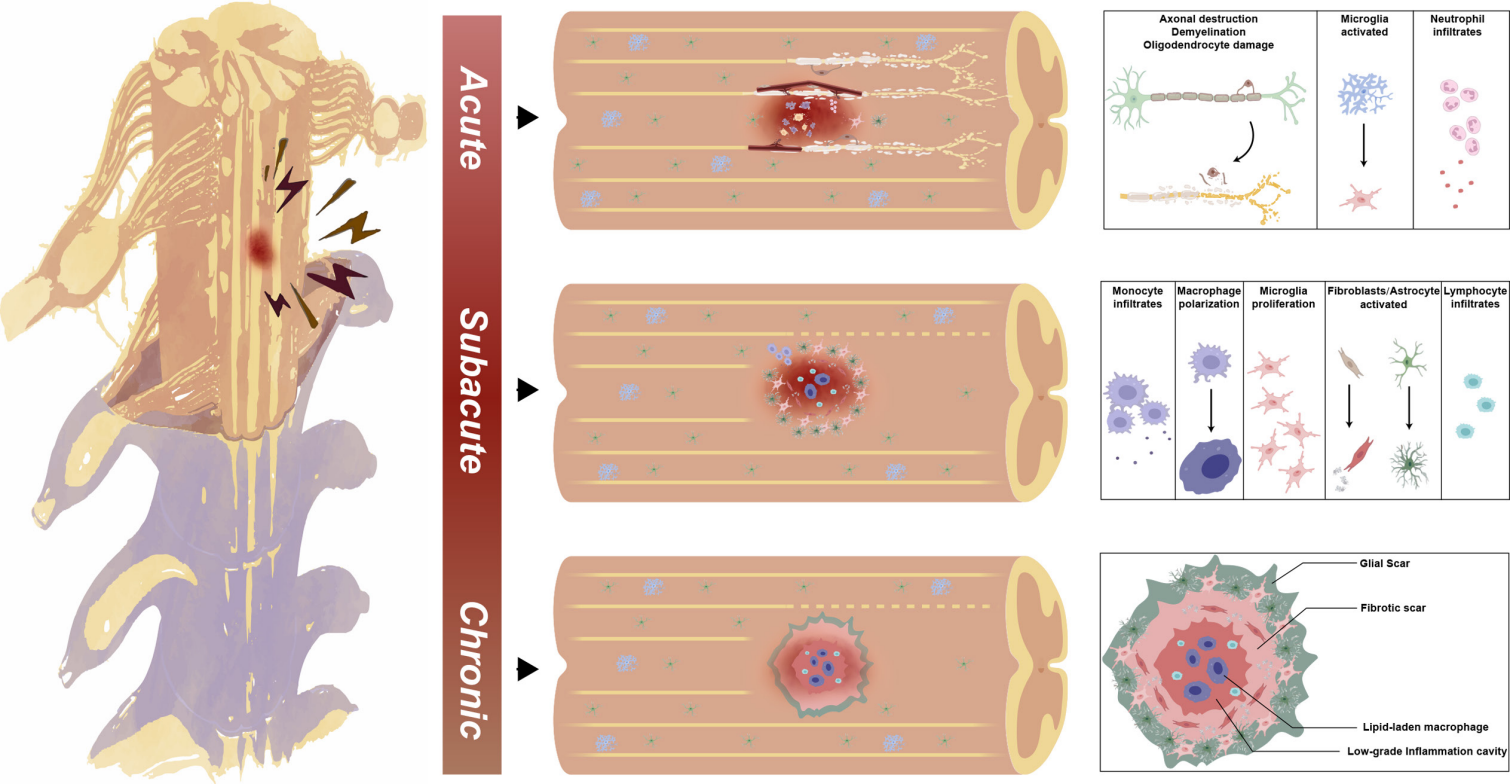
Three stages of SCI—acute (< 48 hours), subacute (48 hours–14 days), and chronic (> 14 days)—along with the key biological events within each stage. The left panel depicts a spinal cord cross-section, with the stages representing the progression over time from the acute stage to the subacute stage and finally the chronic stage labeled from top to bottom. The acute phase represents the initial stage of SCI. Arrows indicate the onset of damage, which is followed by axonal loss, oligodendrocyte destruction, and an inflammatory response. The subacute phase constitutes the changes occurring at the injury site over time. The diagram shows monocyte infiltration, macrophage activation, and gliosis. This period also involves the activation of fibroblasts and astrocytes. The chronic phase is characterized mainly by the outcomes of chronic inflammation. The diagram shows the presence of glial scars, fibrotic scars, and lipid-laden macrophages. In the chronic phase, prolonged inflammation can lead to the formation of a cavity. The right side of the diagram provides illustrations of the specific biological processes and cell types involved in each stage. Each stage includes corresponding processes, such as axonal demyelination, oligodendrocyte damage, and microglial activation in the acute phase. The subacute phase is characterized by monocyte and macrophage infiltration, as well as microglial proliferation. The chronic phase is characterized by the formation of a glial and fibrotic scar. SCI: Spinal cord injury.

Secondary injury begins almost immediately as biochemical cascades are activated within neural and vascular tissues. This phase is typically divided into acute (< 48 hours), subacute (48 hours to 14 days), and chronic (> 14 days) stages (Ahuja et al., 2017; Guo et al., 2021). Within minutes of the initial trauma, a prolonged “secondary inflammatory cascade” is initiated, progressively damaging intact tissue and destroying surviving neurons. The processes driving secondary injury include vascular compromise, ischemia, edema, excitotoxicity, electrolyte disturbances, oxidative stress, and apoptosis. Although each stage of secondary injury is associated with distinct pathophysiological events, the transitions between stages are not sharply defined and are generally classified by approximate time frames rather than definitive biological markers (Ortega et al., 2023). In the sections that follow, we examine the spatiotemporal patterns of the cellular components involved in the secondary injury across these pathological stages (**Additional Table 1** and **Figure 1**).

**Additional Table 1:** Summary of spatiotemporal disruption of the inflammatory microenvironment (STDIM)

Phase	Time range	Main cell types	Microenvironmental features
**Acute phase**	< 48 h	- Oligodendrocytes - Microglia - Astrocytes - Neutrophils	(1) Inflammation initiation: Pro-inflammatory cytokines (IL-1β, TNF-α, IL-6) increase rapidly, with IL-6 mRNA expression peaking within 1 h and decreasing after 24 h. (2) Blood-spinal cord barrier disruption: Microvascular rupture leads to hemorrhage and ischemia. Neutrophils infiltrate and release ROS and proteases, exacerbating tissue damage. (3) Cell death: Oligodendrocytes die due to mechanical injury, ischemia, and glutamate excitotoxicity, resulting in demyelination. (4) Microglial activation: They quickly migrate to the injury site, phagocytose debris, and release chemokines (such as CXCL1) to recruit peripheral immune cells.
**Subacute phase**	48 h-14 d	- Monocytes/macrophages - Microglia - Fibroblasts - Astrocytes - Lymphocytes	(1) Immune cell transition: After neutrophils undergo apoptosis, monocytes are recruited and differentiate into macrophages (M1 type is pro-inflammatory, M2 type is anti-inflammatory). There are foam-like macrophages (enriched in lipid metabolism-related genes). (2) Scar formation: Fibroblasts proliferate and secrete ECM to form fibrotic scars; astrocytes gather to form glial scars, and high expression of CSPGs inhibits axonal regeneration. (3) Lymphocyte infiltration: T cells and B cells persist, exacerbating inflammation through cytokines such as IFN-γ. CD8^+^ T cells damage the blood-spinal cord barrier.
**Chronic phase**	>14 d	- Microglia/macrophages - Fibroblasts - Astrocytes - Lymphocytes	(1) Low-level chronic inflammation: Immune cells (such as lipid-laden macrophages) exist for a long time, maintaining an inflammatory state and hindering repair. (2) Scar stabilization: The structures of glial scars and fibrotic scars are solidified, becoming physical and biochemical barriers to axonal regeneration. (3) Stagnation of tissue remodeling: Fibroblasts and astrocytes stop migrating, and ECM (such as collagen) is excessively deposited, forming cavities and fibrotic areas.

CSPGs: Chondroitin sulfate proteoglycans; CXCL1: C-X-C motif chemokine ligand 1; ECM: extracellular matrix; IFN-γ: interferon-y; IL-1β: interleukin-1β; IL-6: interleukin-6; ROS: reactive oxygen species; STDIM: Spatiotemporal disruption of the inflammatory microenvironment; TNF-α: tumor necrosis factor-α.

#### Temporal–spatial dynamics of inflammatory responses across disease stages

(1) Acute phase (< 48 hours)

• Oligodendrocytes

During the acute phase, bleeding and neurogenic shock resulting from the primary injury lead to hypovolemia and hemodynamic alterations, affecting blood perfusion in the spinal cord and causing localized ischemia. Although larger vessels, such as the anterior spinal artery, generally remain intact, smaller intramedullary blood vessels and capillaries are prone to damage, leading to the extravasation of white blood cells and red blood cells (Furlan and Fehlings, 2008). Cells subjected to severe trauma undergo necrosis and fragmentation, exhibiting signs of shrinkage, pyknosis, and compaction, along with mitochondrial and plasma membrane swelling, indicating irreversible damage (Crowe et al., 1997). In this environment, the oligodendrocytes that ensheath axons are among the first to be impacted, leading to demyelination and cell death (Duncan et al., 2020). Causes of oligodendrocyte death include mechanical trauma, ischemia, hypoxia, and excitotoxicity from glutamate and ATP. Proteolytic enzymes released by necrotic cells or damaged blood vessels also contribute to the further degradation of cellular and vascular structures, exacerbating injury. Ischemia and reperfusion additionally generate free radicals, such as reactive oxygen and nitrogen species, which trigger oxidative stress and damage cell membranes, proteins, and DNA (Almad et al., 2011; Alizadeh and Karimi-Abdolrezaee, 2016).

Oligodendrocytes are highly susceptible to damage during the acute phase of SCI due to their elevated metabolic demands, high intracellular iron content (Cheli et al., 2020; Huang et al., 2025), and comparatively weak antioxidant defenses. Excitotoxicity is a central contributor to their death (Nasrabady et al., 2018). Following injury, excessive glutamate and ATP levels overstimulate alpha-amino-3-hydroxy-5-methyl-4-isoxazolepropionic acid (AMPA), N-methyl-D-aspartate (NMDA), and P2X7 receptors, driving calcium overload, mitochondrial dysfunction, and ultimately cell death (Jia et al., 2016; Maher et al., 2018). These receptor-mediated events activate a range of signaling cascades, including the calcium/calmodulin-dependent protein kinase II (CaMKII) and c-Jun N-terminal kinase (JNK)/p38 mitogen-activated protein kinase (MAPK) pathways, which mediate apoptosis and necroptosis (Quintela-Lopez et al., 2019; Marziali et al., 2024), as well as nuclear factor kappa-light-chain-enhancer of activated B cells (NF-κB) and nuclear factor erythroid 2-related factor 2 (Nrf2) responses triggered by oxidative stress. NF-κB activation increases pro-inflammatory gene expression, whereas Nrf2-induced antioxidant defenses—although protective—are often inadequate in oligodendrocytes (Fadoul et al., 2024; Navarro and Esteras, 2024; Shen et al., 2024). The convergence of these processes explains why oligodendrocytes are among the earliest and most extensively affected cell types after SCI.

• Microglia

Following SCI, myelin breakdown and cellular debris activate microglia, the resident immune cells in the CNS (Brockie et al., 2021). In their resting state, microglia have many tiny, fuzzy protrusions that constantly survey their environment. When they encounter an injury signal nearby, they slowly move toward it at a rate of approximately one inch every minute and a half (David and Kroner, 2011). Upon arrival at the injury site, they transition into amoebic phagocytes that consume and destroy cellular and myelin debris, resulting in the development of a proinflammatory microenvironment (Ransohoff and Perry, 2009). Activated microglia also secrete chemokines to attract immune cells from the periphery, helping to form an early, tight barrier of cells that appears to restrain the lesion and prevent further spread. This boundary is established by functional chloride (Cl^–^) channels and modulated by ATP, G-protein-coupled purinergic P2Y12 receptors, outward K^+^ currents, connexin hemichannels, integrin β1, and downstream phosphoinositide 3-kinase (PI3K)/Akt signaling (Ohsawa et al., 2010; Bellver-Landete et al., 2019). Taken together, these findings demonstrate that the myriad regulatory elements for microglial motility at the site of injury are indeed hallmarks of the earliest events in SCI.

*In vivo* imaging studies using two-photon laser scanning microscopy confirm that ATP and nitric oxide act as key chemotactic signals that guide microglial migration and process extension toward injury sites (Huang et al., 2021; Wu et al., 2024). Once activated, microglia upregulate proinflammatory mediators such as IL-1β and TNF-α, which contribute to bystander cytotoxicity in neurons and oligodendrocytes, amplifying secondary injury (Ferguson et al., 2008; David and Kroner, 2011). Moreover, microglia generate peroxynitrite, a highly reactive oxidant formed by the combination of nitric oxide and superoxide, which induces neuronal death (Wilkinson and Landreth, 2006). These processes are mediated by key inflammatory signaling pathways, such as the NF-κB pathway, which drives the transcription of IL-1β, TNF-α, and iNOS, as well as the MAPK family (particularly p38 and JNK), to further promote microglial activation and cytokine production. The excessive generation of ROS and RNS by activated microglia exacerbates oxidative stress, disrupts cellular homeostasis, and impedes tissue repair (Kraft and Harry, 2011; Shao et al., 2022).

• Astrocytes

Classically activated microglia induce reactive astrocytes to polarize toward a proinflammatory phenotype characterized by increased expression of various complement proteins, such as C1r, C1s, C3, and C4, along with the proinflammatory factors IL-1β, TNF-α, and nitric oxide (Liu et al., 2020b). Glial fibrillary acidic protein expression increases within hours of SCI. However, morphologically, most reactive astrocytes appear similar to their normal counterparts (Stukas et al., 2023).

Astrocytes can be roughly divided into two types of cells: protoplasmic and fibrous astrocytes. Protoplasmic astrocytes reside in gray matter. Their short, highly branched processes form networks around neurons and synapses that support the structure of synapses. Fibrous astrocytes are located in the white matter area and are relatively large, with long fibers that are aligned along nerve fibers (Wang et al., 2024b). After SCI, astrocytes transition to a proinflammatory state, lose their homeostatic function and participate in secondary damage. This pathological switch is controlled by many signaling pathways. NF-κB signaling, triggered by molecules such as IL-1 and TNF-α, intensifies inflammation since it causes the generation of more cytokines and extra complement elements (Pathak and Sriram, 2023). Moreover, TLR‒MyD88 signaling induced by damage-associated molecular patterns (DAMPs) also promotes the activation of NF-κB and stimulates the activation of the MAPK pathway, including JNK and p38. It also sustains proinflammatory gene expression (Ageeva et al., 2024; Bhatt et al., 2024). Furthermore, JAK/STAT signaling is frequently induced by IL-6 family cytokines such as LIF and CNTF and promotes the proliferation of astrocytes and the development of glial scars (Zhang et al., 2021b; Zhou et al., 2023).

• Neutrophils

Activated microglia and astrocytes release proinflammatory cytokines and chemokines, including IL-1a, IL-1β, IL-8, TNF-α, granulocyte colony-stimulating factor, CCL3, CXCL1, CXCL2, and CXCL5 (Choi et al., 2014; Hong et al., 2018). Additionally, complement activation and activation of the NF-κB signaling pathway attract neutrophils to the injury site; thus, neutrophils are the first immune cells to infiltrate the damaged area (Kitade et al., 2023). IL-1β is a critical initial proinflammatory cytokine produced after injury. It is released by astrocytes and microglia and reaches peak expression within 12 hours of injury, which is correlated with the peak infiltration of neutrophils in the damaged region (Neirinckx et al., 2014).

Upon arriving at the lesion core, neutrophils contribute to secondary tissue damage through the release of proteolytic enzymes (e.g., elastase), oxidases, and tissue-degrading factors. Mechanistically, this cytotoxic response involves the activation of nicotinamide adenine dinucleotide phosphate (NADPH) oxidase, leading to the generation of ROS and increased matrix metalloproteinase (MMP)-9 activity, which disrupts the blood‒spinal cord barrier and promotes extracellular matrix (ECM) degradation. TNF-α released by neutrophils further amplifies inflammation via TNFR1–NF-κB signaling, exacerbating neuronal and glial cell injury. Despite their traditionally deleterious role, neutrophils may also contribute to the early phases of inflammation resolution and tissue remodeling (Nguyen et al., 2007). This dual role is partly mediated by their timely apoptosis and subsequent clearance by macrophages through efferocytosis, which may induce the anti-inflammatory reprogramming of the local immune environment (Ghasemlou et al., 2010; Schreiber et al., 2013).

**Figure 2:**
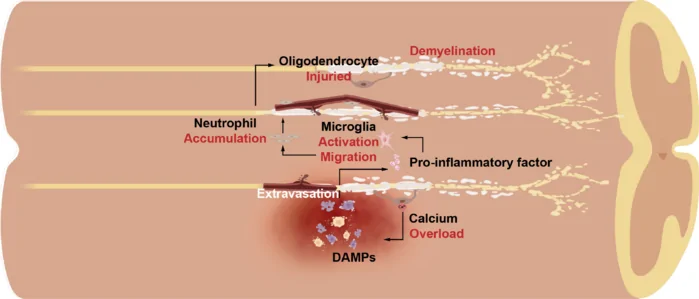
Spatiotemporal characteristics of the inflammatory microenvironment during the acute phase of spinal cord injury. Primary mechanical shock occurs immediately after the injury, after which the blood–spinal cord barrier is disrupted, and the extracellular fluid leaks into the spinal cord. Necrotic cells release DAMPs, which activate microglia and guide microglial movement toward the lesion core. Early inflammatory reactions can attract a large number of proinflammatory immune cells, mainly neutrophils. Moreover, membrane injury and dysregulation of ion channels lead to calcium overload in cells and aggravate oligodendrocyte injury. All these factors cause demyelination, worsening secondary tissue injury and maintaining an unfriendly environment that prevents repair. DAMPs: Damage-associated molecular patterns.

Primary mechanical shock occurs immediately after the injury, after which the blood–spinal cord barrier is disrupted, and the extracellular fluid leaks into the spinal cord. Necrotic cells release DAMPs, which activate microglia and guide microglial movement toward the lesion core. Early inflammatory reactions can attract a large number of proinflammatory immune cells, mainly neutrophils. Moreover, membrane injury and dysregulation of ion channels lead to calcium overload in cells and aggravate oligodendrocyte injury. All these factors cause demyelination, worsening secondary tissue injury and maintaining an unfriendly environment that prevents repair (**Figure 2**).

(2) Subacute phase (48 hours–14 days)

• Monocytes and macrophages

Neutrophils, short-lived cells of the innate immune system, play roles in degranulation, phagocytosis, and the formation of neutrophil extracellular traps during the subacute phase of SCI. Following these activities, neutrophils undergo programmed cell death, releasing matrix metalloproteinases and chemotactic signals that disrupt the integrity of the blood–brain barrier and aid in the recruitment of monocytes to the damaged region. Monocytes are subsequently recruited to the site through chemotactic signals, such as MCP-1. Once at the injury site, monocytes differentiate into macrophages in response to the cytokines and chemokines present in the pathological environment and begin phagocytosing dying neutrophils (Yokota et al., 2017).

This phagocytic process involves the interaction of the scavenger receptor CD36 with the vitronectin receptor and thrombospondin-1, while other “eat-me” signals, such as phosphatidylserine or extracellular ATP, are recognized by receptors such as Mertk (a TAM family receptor) and P2Y receptors, thereby activating the PI3K/Akt and Rac1 signaling pathways that promote cytoskeletal remodeling for engulfment (Nagata et al., 2016). As macrophages accumulate, they adopt context-dependent phenotypes. Classically activated M1 macrophages exhibit a proinflammatory profile driven by TLR–MyD88–NF-κB and STAT1 signaling in response to IFN-γ and lipopolysaccharide (LPS), and produce high levels of TNF-α, IL-1β, ROS, and degradative enzymes (e.g., collagenases and furins), thereby exacerbating tissue damage and impairing regeneration (Fan et al., 2016).

However, M2 macrophages are activated through the IL-4/IL-13–STAT6 signaling pathway, which induces the production of anti-inflammatory proteins such as Arg1, IL-10, and transforming growth factor-beta (TGF-β). These proteins contribute to ECM remodeling, promote axonal regeneration, and promote the proliferation of stem and progenitor cells (Nakazaki et al., 2021; Zhang et al., 2021a). As has become increasingly evident, the conventional M1/M2 dichotomy is overly simplistic, especially within a living body. Recently completed transcriptomic studies have identified hybrid/transitional phenotypes of “foam-like” macrophages with increased lipid metabolism and phagocytic gene expression. Such populations could be integral to fine-tuning the interplay between inflammation and tissue repair (Kong et al., 2020; Van Broeckhoven et al., 2021). These findings suggest that the function of macrophages in SCI should follow a dynamic process influenced by both spatiotemporal cues and integrated signaling pathways.

• Microglia

During the subacute phase of SCI, microglia proliferate extensively, and many amoeboid, phagocytotic microglial cells cluster at the edge of the spinal cord lesion. Within the lesion center, large and round “foam-like” microglia with lipid droplets are often observed, whereas highly branched, ramified microglia remain in the spared gray matter and distal white matter (Gao et al., 2023; Sun et al., 2023a). This particular way of spatial organization can reveal the functional differences between different types of microglia and nearby environments. Emerging evidence indicates that the deletion of TREM2 shifts microglia toward a predominantly purinergic sensing state, representing a transition from active phagocytosis to increased purinergic surveillance (Gao et al., 2023; Zhao et al., 2025). This state is characterized by increased expression of the P2Y12 receptor, which reprograms microglia to increase their sensitivity to extracellular ATP and promotes a reparative response resembling the scar-free recovery observed in neonatal SCI models (Li et al., 2020; Gao et al., 2023). Mechanistically, TREM2 signals through the DAP12–SYK pathway to facilitate phagocytosis, lipid processing, and protection from cell death. The loss of TREM2 downregulates the expression of phagocytic genes such as Apoe, Tyrobp, and Lpl and may enhance the purinergic surveillance mediated by PI3K/Akt–Rho GTPase–cytoskeletal remodeling (Wang et al., 2021a; Zhang et al., 2025c; Zhao et al., 2025). However, the indiscriminate elimination of microglia, such as through CSF1R inhibition, interferes with clearing away debris, alters how glial scars form, and prevents the restoration of nerve growth (Brennan et al., 2022). Microglial phagocytic activity is necessary for removing myelin and cellular debris and controlling excessive neuronal excitability, yet both overstimulation and exhaustion aggravate tissue damage (St-Pierre et al., 2023).

• Fibroblasts

Macrophages and microglia contribute extensively to the fibrotic response following SCI through the release of numerous pro-fibrotic cytokines and growth factors, including TGF-β1, PDGF, CTGF, and FGF-2 (Ayazi et al., 2022). These cytokines and growth factors directly activate fibroblasts, leading to their proliferation, maturation, and the production of many ECM components. Under normal conditions, few fibroblasts are present in the perivascular region of the spinal cord. However, following injury, they rapidly expand and become the primary drivers of neuroinflammation and fibrotic scarring (Munk et al., 2019). Approximately 3 days after injury, fibroblasts begin to multiply and migrate into the lesion center, reaching their peak quantity by day seven. At this time, a large amount of fibronectin is deposited, forming a scaffold as a scar begins to develop.

The activation and proliferation of fibroblasts are guided by various interconnected signaling pathways. TGF-β1 activates the Smad2/3 pathway, leading to fibroblast/myofibroblast transformation and collagen synthesis (Deng et al., 2024; Wang et al., 2024a). PDGF–PDGFR-β signaling increases fibroblast proliferation and chemotaxis through PI3K–Akt activation (Yun et al., 2025). CTGF, a downstream effector of TGF-β and PDGF, enhances ECM production and stiffening in tissues (Li et al., 2022c; Holl et al., 2024; Sun et al., 2025). Additionally, it can stimulate cell cycle re-entry and ECM remodeling via the ERK1/2 pathway (Lee et al., 2023).

In the subacute phase, lipid-rich fatty macrophages are observed around the edges of the lesion. They collaborate with fibroblasts to solidify the fibrotic matrix into a large scar. On one hand, this scar forms a barrier that restricts the further spread of inflammation and prevents additional deterioration of the tissue. On the other hand, it acts as both a physical and chemical barrier, preventing neural stem/progenitor cells from infiltrating deeper into the lesion core and hindering axon regrowth (Li et al., 2021; Dorrier et al., 2022). If all scar-associated fibrosis could be fully reversed, such as by genetically eliminating the core fibroblast effectors, the lesion would destabilize and a cavity would form. Alternatively, partial modulation of scar formation—such as fibroblast-specific deletion of HRas, NRas, and KRas, which suppressed fibroblast proliferation through RAS-dependent inhibition of mitogenic signaling—also improved function in mouse SCI models without compromising lesion stability (Dias et al., 2018). These results highlight the importance of therapeutic approaches that balance the induction of pro-regenerative tissue remodeling with the maintenance of structural stability.

• Astrocytes

The responsiveness of astrocytes to microglia and macrophages increases during this phase. At 3–5 days post-SCI, there is an explosive increase in the number of astrocytes, which accumulate around the injury site to form a glial scar with extended web-like processes (Kong et al., 2020). Additionally, it is important to note that the glial scar exhibits a spatially directed organization. Magnetic resonance imaging has revealed that the average thickness of the glial scar on the rostral/caudal side is greater than that on the left/right side of the rat spinal cord cavity (Telegin et al., 2022). One of the most significant features of the glial scar is the increased expression of ECM components, which are primarily secreted by reactive astrocytes. Among these components, chondroitin sulfate proteoglycans (CSPGs) are likely the best characterized due to their inhibitory effects on axonal regeneration and neuroplasticity. Both *in vitro* and *in vivo* studies have demonstrated that CSPGs block axonal extension primarily by activating RhoA/ROCK, which disrupts the cytoskeleton and prevents the forward migration of growing nerve fibers (Stern et al., 2021). The JAK/STAT3 and NF-κB pathways regulate CSPG transcription, with STAT3 playing a key role in astrocyte proliferation, process extension, and glial scar assembly (Pan et al., 2021). Conditional deletion of STAT3 in astrocytes disrupts scar architecture and exacerbates axonal dieback (Wanner et al., 2013; Liu et al., 2024a).

Astrocytes also display unique spatial and morphological patterns during glial scar formation (Schachtrup et al., 2010). Initially, astrocytic processes point toward the lesion center and extend deep into it, where new, migrating astrocytes accumulate and adopt similar forms. Approximately 2 weeks after injury, the scar structure matures; the processes of scar-forming astrocytes no longer extend to the center of the lesion but instead run in parallel lines and overlap. Meanwhile, reactive astrocytes located further away from the injury site gradually revert to their quiescent form and restore homeostatic function (Hara et al., 2017). This difference in astrocyte shape and position does not appear to be entirely dependent on their spatial location. Understanding how these glial cell scars form and subsequently affect neuronal repair in the spinal cord is crucial.

• Lymphocytes

In the subacute phase of SCI, both fibrotic scars and glial scars work together to exert protective effects by limiting excessive inflammation associated with lymphocyte infiltration (Perez-Gianmarco and Kukley, 2023). Experiments have shown that the selective knockout of proliferating reactive astrocytes that participate in scarring, such as through the deletion of Stat3, dramatically increases the infiltration of monocytes, myelomonocytic cells, neutrophils, and lymphocytes by approximately 25-fold (Okada et al., 2006). Large-scale infiltration of these adaptive immune cells is limited because T and B lymphocytes induce central nervous system-specific autoimmune responses that persist long after SCI, directly causing neuronal and glial cell death (Jones, 2014).

Mechanistically, CD4^+^ T cells contribute to inflammation via the TCR–NFAT and IFN-γ–STAT1 signaling pathways, producing pro-inflammatory cytokines such as IL-2, IFN-γ, and TNF-α (Zhong et al., 2025). Moreover, γδT cells also serve as early sources of IFN-γ, further amplifying inflammation during SCI (Sun et al., 2018b). Evidence from SCI models with T-cell and B-cell deficiency revealed improved neurological recovery and confirmed the role of adaptive immune responses in pathogenesis (Liu et al., 2019b). Additionally, perforin from CD8^+^ T cells damages the blood–spinal cord barrier, allowing more inflammatory cells and cytokines to enter (Liu et al., 2019b). T-cell infiltration and signaling in the adult dorsal spinal cord are also associated with the development of neuropathic pain-like hypersensitivity (Costigan et al., 2009).

**Figure 3:**
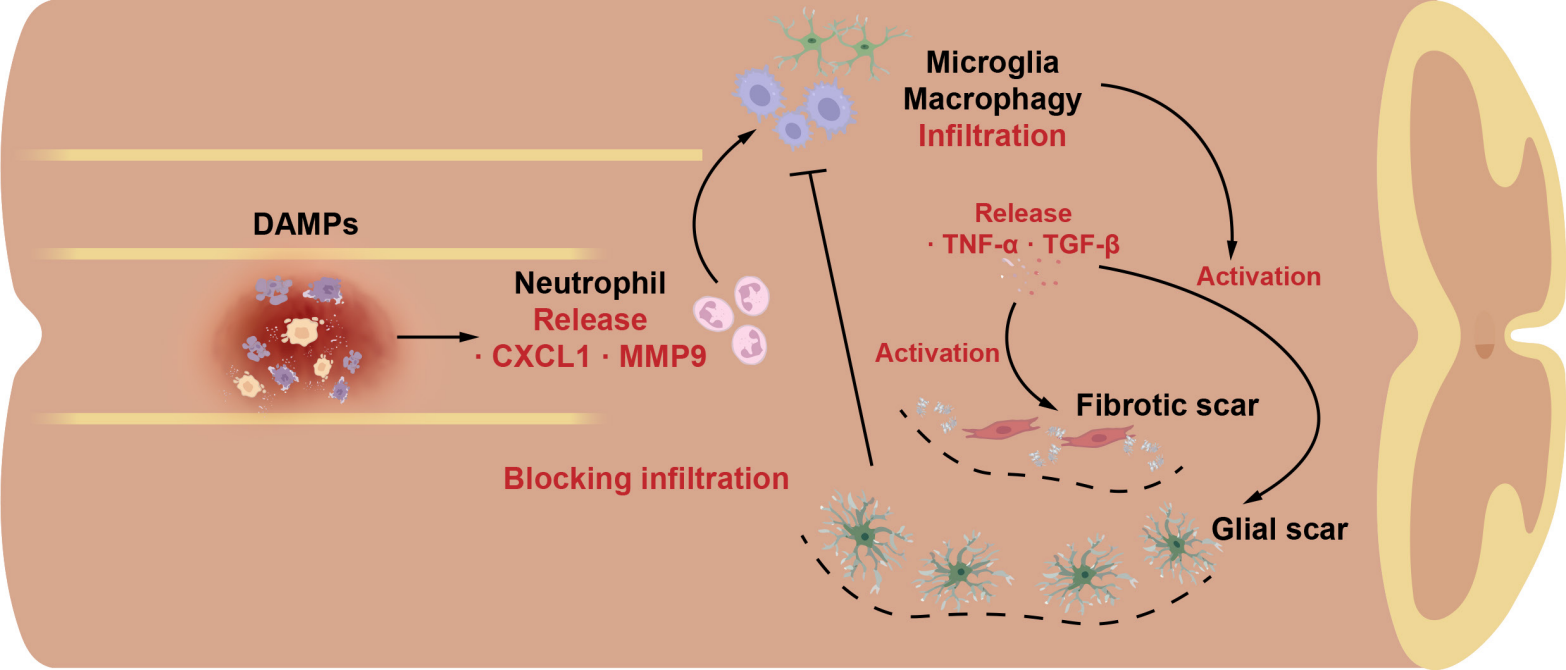
Spatiotemporal characteristics of the inflammatory microenvironment during the subacute phase of spinal cord injury. CXCL1: C–X–C motif chemokine ligand 1; DAMPs: damage-associated molecular patterns; MMP9: matrix metalloproteinase 9; TGF-β: transforming growth factor-beta; TNF-α: tumor necrosis factor-alpha.

During the subacute stage, immune cell infiltration into the lesion continues. Neutrophils continuously secrete chemokines such as C–X–C motif chemokine ligand 1 (CXCL1) and enzymes such as matrix metalloproteinase (MMP) 9, which help combat inflammation. Activated microglia and infiltrating macrophages clear debris; at the same time, these immune cells secrete cytokines such as TNF-α and TGF-β, which exert downstream effects. They stimulate astrocytes and fibroblasts to generate a scar comprising both gliotic and fibrotic components. This composite scar serves a dual purpose as a physical and biochemical barrier; it effectively prevents further infiltration of immune cells into the lesion while also creating a formidable impediment to axonal regrowth (**Figure 3**).

(3) Chronic phase (> 14 days)

• A persistent low-grade inflammatory milieu

The typical scarless healing process in normal tissue injury involves several stages. It begins with the formation of a platelet-induced fibrin clot that stops bleeding while providing a scaffold for cells and growth factors to fill the early wound bed. This step is followed by the inflammatory phase, involving early responders such as mast cells, neutrophils, and inflammatory macrophages, which are tasked with infection prevention and debris phagocytosis. These cells also release signaling molecules that coordinate the healing process. The proliferative phase includes angiogenesis, the replenishment of reparative macrophages, and the activation of resident cells in adjacent healthy tissue to divide and migrate into the wound. Organs with epithelial cells undergo re-epithelialization, and fibroblasts migrate into the provisional ECM, where they produce collagen, which is then aligned and organized into scar tissue during the remodeling stage. Macrophages reduce the volume of the wound bed and remove excess ECM through degradation, leading to scarless healing, with most of the original tissue structure and function restored as macrophages and fibroblasts undergo apoptosis and are cleared (Younesi et al., 2024).

However, unlike successful wound healing, SCI becomes trapped in a state of STDIM. Microglia and monocyte-derived macrophages persist indefinitely within the lesion core, maintaining an activated state without undergoing the usual temporal shift from proinflammatory to prorepair processes characteristic of scarless healing (Silver et al., 2014). In addition, because the spinal cord ECM contains fewer collagen components, the collagen produced by fibroblasts forms fibrotic scars that hinder axonal regeneration (Kumar et al., 2022). The migration and remodeling of fibroblasts and astrocytes cease, reinforcing the structure of the glial and fibrotic scars (Bradbury and Burnside, 2019). The acquired immune system, with lymphocytes infiltrating and remaining indefinitely in the scar, creates a low-level inflammatory environment that can persist for years. This chronic inflammation, which is maintained by the constant presence of immune cells, significantly hinders neural repair and recovery (Allison and Ditor, 2015).

**Figure 4:**
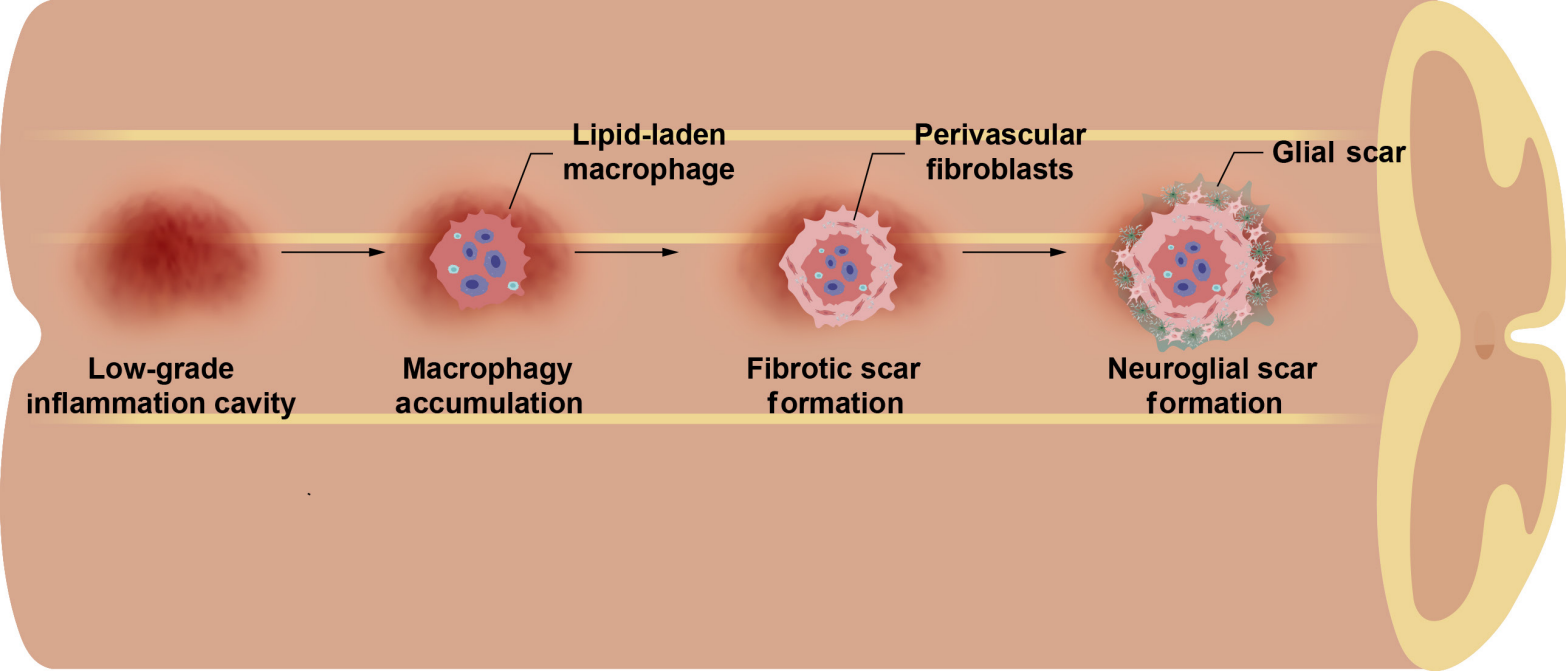
Spatiotemporal characteristics of the inflammatory microenvironment in the chronic phase of spinal cord injury. In the chronic phase, the lesion site exhibits a highly organized and compartmentalized structure. At the center, there is a cavitation characterized by low-grade, ongoing inflammation, with lipid-laden macrophages recruited by pro-inflammatory signaling, which impede repair processes. The central cavity is primarily composed of a dense fibrotic scar made up of perivascular fibroblasts and an abundance of extracellular matrix. Surrounding the fibrotic layer are glial scars, mainly composed of reactive astrocytes and microglia. This spatial structure acts as a barrier, preventing immune cell infiltration and hindering axonal regrowth, ultimately leading to long-term neurological deficits.

Site has a very organized and compartmentalized structure. A cavitation with low-grade, ongoing inflammation is present at the center, with lipid-laden macrophages recruited by pro-inflammatory signaling and impeding repairs. The central cavity is composed mainly of a dense fibrotic scar composed of perivascular fibroblasts and a large amount of matrix. Glial scars form around the fibrotic layer, which is composed mainly of reactive astrocytes and microglia. It is a spatial structure of a cell, and then, it serves as a barrier to immune cells out and prevents axonal regrowth, leading to long-term neurological deficits (**Figure 4**).

### Emerging methodologies for mapping and modeling the inflammatory microenvironment

In recent years, the rapid advancement of spatiotemporal transcriptomics, AI-driven drug screening, and organoid modeling has provided powerful tools for systematically dissecting the composition and dynamic changes in the inflammatory microenvironment during SCI. In developmental studies (Andersen et al., 2023; Li et al., 2023b), Shi et al. (2024) used a combination of single-cell RNA sequencing, TF-seq, FISH, and 10× Visium spatial transcriptomics to construct a high-resolution transcriptional atlas of the human embryonic spinal cord from gestational weeks 7 to 25. Their findings reveal the precise dorsoventral and mediolateral organization of neural cells and clarify the transcription factors governing these spatial patterns. Importantly, Shi et al. (2024) identified a distinct microglial subset with heightened vulnerability to pathological states, providing new perspectives on spinal cord–related diseases.

Gong et al. (2023) utilized spatial transcriptomics to generate a detailed molecular atlas of scar tissue following SCI in experimental injury models, pinpointing neurotoxic lipid accumulation, ECM remodeling, and immune activation as central drivers of scar formation and regeneration failure. Building on this information, Skinnider et al. (2024) created “Tabulae Paralytica,” which combines more than 500,000 single-nucleus transcriptomes with multiomic and time-resolved spatial transcriptomic data. This comprehensive atlas documents major pathological processes, including changing cell states, abnormal brain circuit formation, failure to restore barrier integrity, and reduced regenerative capacity with age, making it the broadest cellular reference chart to date related to a single case of SCI (Skinnider et al., 2024). Moreover, Wang et al. (2024b) used spatial multiomics to construct stage-specific gene expression programs and migration trajectories of different cell types in a mouse model of complete spinal transection. They reported that a subset of astrocytes migrated to discrete regions, suggesting structural repair by these cells.

In addition to advances in spatial omics, tissue-engineered and organoid-based strategies have shown substantial regenerative potential. Spinal cord–like tissues designed to reproduce the native spinal cord architecture and lineage diversity have been shown to support axonal elongation, synapse formation, and integration into host neural networks. Wertheim et al. (2022) demonstrated that patient-derived induced pluripotent stem cell-based spinal cord-like tissues embedded in biocompatible hydrogels markedly improved motor function in chronic SCI models. Lai et al. (2023) designed modular spinal cord-like tissues that replicated the structural distinction between white and gray matter, enabling complex circuit reconstruction after transplantation; their therapeutic benefit was further enhanced when combined with caudal nerve electrical stimulation. Xu et al. (2023) reprogrammed human astrocytes into neuroectoderm-like cells to generate spatially patterned spinal organoids with dorsal or ventral specificity. These organoids successfully established synaptic connectivity with host neurons and bridged lesion gaps in experimental SCI models, highlighting their translational promise.

In summary, spatial transcriptomics has become an indispensable tool for mapping the temporal and anatomical complexity of SCI, enabling the precise identification of cell populations and molecular pathways that shape disease progression. Organoid-based systems offer physiologically relevant, structurally organized tissue substitutes for neural repair. The combination of these technologies could pave the way for the development of highly targeted, spatiotemporally optimized regenerative interventions.

## Multimodal Intervention Strategies for Modulating the Inflammatory Microenvironment in Neurotrauma, Neurodegeneration, and Related Disorders

Correcting the STDIM following SCI and establishing an organized environment for regeneration and repair are crucial for the restoration of neurological function. Currently, a variety of promising therapeutic strategies in which the spatiotemporal targets of inflammatory cells are directly targeted to promote nerve repair and regeneration are emerging.

### Stem cell–based regenerative and immunomodulatory therapies

Mesenchymal stem cells (MSCs) show significant potential for treating SCI because of their comprehensive biological functions, such as promoting angiogenesis and axonal regeneration, inhibiting neuronal apoptosis, limiting lesion progression, reducing glial scarring, and mitigating inflammatory responses (Assinck et al., 2017). MSCs intricately modulate the immune response through the secretion of cytokines and growth factors, suppress fibrosis and apoptosis, promote angiogenesis, activate endogenous stem cells, and actively participate in the remodeling of the cellular microenvironment, thus creating favorable conditions for spinal cord repair (Andrzejewska et al., 2021). In particular, human umbilical cord mesenchymal stem cells (hUCMSCs) are a focal point of research because of their rapid proliferation, low immunogenicity, exceptional regenerative and adaptive capabilities, multidirectional differentiation and anti-inflammatory properties, as well as their accessibility and minimal ethical controversies (Yang et al., 2020a, b; Lu et al., 2022; Ribeiro et al., 2023). Numerous studies have indicated that timely transplantation of hUCMSCs significantly enhances neurological function recovery, positively affecting the treatment of SCI (Yang et al., 2021; Cao et al., 2022; Lu et al., 2022). Currently, therapeutic strategies for SCI primarily focus on controlling secondary damage, with the goal of increasing treatment efficacy by suppressing neuroinflammation and fostering an environment conducive to neurogenesis and axonal regeneration (Zhang et al., 2025a). Despite some progress with existing methods, curing SCI remains a significant challenge. hUCMSCs comprehensively modulate postinjury immune and inflammatory responses by directly interacting with microglia and astrocytes and regulating peripheral immune cells and immune organs. In particular, the MAPK and NF-κB signaling pathways are considered major pathways controlling the response to inflammation in SCI, and its unusual excitation can induce excessive production of inflammatory cytokines (Qian et al., 2020). hUCMSCs reduce local inflammation by preventing MAPK activation after injury, decreasing neuronal apoptosis, and decreasing the levels of proinflammatory cytokines such as IL-6, IL-7, IL-8, IFN-γ, IL-1β, and TNF-α (Sun et al., 2018a). Furthermore, microglia, which are primary innate immune cells in the central nervous system, are pivotal for neuroinflammation resulting from SCI (Shu et al., 2020). During this process, the activation of inflammasomes is part of the initial response, especially the specific expression of the NLRP3 inflammasome in microglia. Na et al. (2020) demonstrated that the local application of hUCMSCs subjected to ultrashort wave treatment could decrease the phosphorylation of protein kinase 2 activated by the MAPK pathway and reduce NLRP3 expression, thereby ameliorating postinjury neuroinflammation. However, further research is needed to understand the mechanisms by which hUCMSCs regulate inflammasomes to guide their clinical application for inflammasome modulation.

Moreover, adipose-derived mesenchymal stem cells (AD-MSCs) are considered good candidates for therapeutic applications because they can be easily harvested from the adipose tissue of patients and possess multipotent characteristics (Bydon et al., 2024). A phase I clinical trial (CELLTOP, NCT03308565) examining the intrathecal administration of autologous AD-MSCs in ten individuals with SCI did not report any serious adverse events during six to nine weeks of follow-up. Seven patients showed some improvement in the American Spinal Injury Association (ASIA) Impairment Scale (AIS), supporting the potential feasibility of AD-MSC-based therapies (Bydon et al., 2024). While MSCs exhibit significant potential for treating SCI, there are several challenges, including poor transplantation ability, rejection by the immune system, risks of genetic changes, the complexity of the procedures involved, and the possibility of tumor growth (Zhang et al., 2013). Additionally, a large glial scar forms following injury, characterized by the overproliferation of glial cells, which severely hinders effective integration, differentiation, and axonal regeneration of MSCs in the lesion area (Cooper et al., 2020).

In recent years, researchers have focused on the paracrine function of MSCs, particularly the secretion of exosomes, which hold special value in the treatment of nervous system diseases, including SCI, due to their high safety, low immunogenicity, long half-life, and ability to cross the blood–brain barrier (Alvarez-Erviti et al., 2011). Exosomes play a crucial role in modulating neuroinflammation. During the secondary injury period of SCI, microglia, neutrophils, macrophages, and lymphocytes are present at the injury site, releasing various pro-inflammatory and anti-inflammatory factors that collectively regulate the intensity and direction of the inflammatory response (Liu et al., 2021). Therefore, controlling the local inflammatory microenvironment is the most effective strategy for treating spinal cord damage.

MSC-derived exosomes improve the postinjury inflammatory environment through the TLR4/MyD88/NF-κB and miR-23b/TLR4/NF-κ pathways (Fan et al., 2021; Hellenbrand et al., 2021; Nie and Jiang, 2021). Inflammasome complexes are also activated during the inflammatory response after SCI, and MSC-derived exosomes promote neuroinflammation and oxidative stress by inhibiting the activity of the microglial NRF2/NF-κB/NLRP3 pathway (Che et al., 2024). Moreover, exosomes modulate macrophage polarization. Macrophages exhibit remarkable heterogeneity and functional plasticity and can be broadly categorized into proinflammatory M1 and anti-inflammatory M2 phenotypes (Jiang et al., 2016). M1 macrophages secrete proinflammatory cytokines, ROS, and NO, thereby exacerbating tissue inflammation and injury; in contrast, M2 macrophages release anti-inflammatory mediators, attenuating inflammatory signaling at the injury site and facilitating tissue repair and remodeling. Macrophage phenotypes are known to shift in response to inflammatory stimuli (Mosser and Edwards, 2008). Research conducted by Sun et al. (2018a) revealed that MSC-derived exosomes could promote the transformation of M1 macrophages to the M2 phenotype. Liu et al. (2020c) found that microRNA 216a-5p carried by exosomes from hypoxia-preconditioned MSCs can control microglial M1/M2 polarization through the TLR4/NF-κB/PI3K/AKT signaling pathway and can facilitate the repair of traumatic SCI. Many studies have highlighted the crucial role of exosomes in injured spinal cord repair; these cells act as key messengers for intercellular communication by transporting bioactive molecules, including proteins, DNA, mRNAs, and miRNAs, that are secreted from the parental cells (Kalluri and LeBleu, 2020). Among these molecules, certain miRNAs have received specific attention because of their regulatory roles in neural recuperation (Cossetti et al., 2014; Sims et al., 2014). Wang et al. (2021b) showed that MSC-derived exosomes carrying miR-199a-3p and miR-145-5p activate NGF/TrkA signaling, markedly improving functional outcomes in a rat SCI model. Similarly, Lai et al. (2022) reported that miR-146a-5p-overexpressing exosomes selectively inhibit neurotoxic astrocytes to promote neuropathological recovery. Xiao et al. (2021) also showed that exosomal delivery of miR-29b-3p activates the PTEN/AKT/mTOR pathway, reduces pathological changes and improves motor function and neural repair in injured rat models.

Targeted miRNAs possess considerable therapeutic potential when applied to SCI, whereas exosomes serve as ideal delivery vehicles that increase biological activity (Wang et al., 2020a, b). For example, miR-124 can help macrophages shift from the proinflammatory M1 phenotype to the anti-inflammatory M2 phenotype, and M2 macrophages with higher miR-124 levels are stabilized and maintain a reparative profile (Ponomarev et al., 2011). Jiang et al. (2020) reported that miR-124-3p and neurogenic exosomes work together to reduce M1 microglial activation via the MYH9/PI3K/AKT/NF-κB pathway, greatly enhancing functional recovery after SCI. A similar function has been observed for miR-146a as an essential modulator of innate immune signaling and neuroinflammation in macrophages, which are associated with various neurological diseases. By directly binding to TRAF6, IRAK1, and IRAK2, it can dampen inflammatory pathways (Chen et al., 2019). In addition, miR-146a-5p suppresses LPS-induced TRAF6 upregulation in the spinal cord through the JNK‒CCL2 pathway to reduce neuroinflammatory damage (Lu et al., 2015). The therapeutic potential of exosomes is directly related to the amount of miR-216a-5p they contain; by blocking the TLR4/NFKB pathway and promoting the PI3K/AKT pathway, microglia transition from the M1 phenotype to the M2 phenotype, with greatly increased therapeutic effects (Xue et al., 2020). Similarly, exosomes derived from MSCs that carry many miRNAs, such as miR-29b-3p, mir-199a-3p, mir-145-5p, and mir-26a, regulate signaling pathways, such as the PTEN/AKT/mTOR and NGF/TrkA pathways, to promote axonal regeneration and improve motor function after SCI (Chen et al., 2021; Xiao et al., 2021). Studies of hUCMSC-derived exosomes have shown that these cells have different functions in response to SCI. CD73-modified hUCMSC-derived exosomes, which activate the A2bR–cAMP–PKA signaling pathway, not only increase inflammation after SCI but also regulate the differentiation of macrophages/microglia from the M1 phenotype to the M2 phenotype (Zhai et al., 2021). Moreover, unmodified hUCMSC-derived exosomes promote the repair of SCI by inhibiting the NF-κB/MAPK and Akt/FoxO3a signaling pathways, thereby decreasing inflammation and neuronal apoptosis (Luan et al., 2024).

In summary, MSCs such as hUCMSCs and their exosomes can serve both as therapeutic agents and as carriers for small molecules such as miRNAs. By precisely regulating multiple signaling pathways and cellular phenotypic transitions, they effectively suppress inflammatory responses, restore neurological functions, and promote tissue regeneration, providing a solid theoretical basis and highly promising therapeutic targets for clinical strategies for the treatment of SCI.

## Bioengineering Approaches: Biomaterials and Tissue Scaffolds for Therapeutic Applications

In recent years, strategies for modulating inflammatory microenvironments have become a hot topic in bioengineering of biomaterials and therapeutic microenvironments (Hu et al., 2023). This section explores the applications of bioengineering techniques in modulating inflammatory microenvironments, particularly how they provide physical support, enable controlled drug delivery and release, coordinate electrical stimulation to promote neural stem cell (NSC) differentiation, and reassemble the ECM and growth factors to regulate the microenvironment, thereby promoting axonal growth and reconstruction of neuronal connections.

### Physical support and controlled drug release

Biomaterials such as hydrogels and conductive scaffolds provide structural support, stabilize damaged areas of the spinal cord, prevent further mechanical damage, and create a repair-friendly environment (Li et al., 2022a; Tian et al., 2025). Hydrogels, in particular, are highly porous three-dimensional matrices with excellent biocompatibility, biodegradability, and mechanical properties that closely resemble native soft tissue. Their structural similarities enable them to provide localized physical support, promoting cell adhesion, growth, differentiation, and tissue regeneration (Wang et al., 2022). Certain biomaterials are engineered with microstructural features or functionalized bioactive ligands that facilitate the adhesion, directed movement, and recruitment of diverse cell types, including neurons, glia, and immune cells. These interactions can alter the phenotype of inflammatory and neuronal cells at the lesion site, generating an environment conducive to regeneration. Notably, some hydrogel components, such as chitosan, hyaluronic acid, and gelatin, possess inherent anti-inflammatory properties, offering additional therapeutic benefits, particularly when inflammation is a major component of the pathophysiology (Hu et al., 2022; Wang et al., 2023).

The three-dimensional network structure and water-holding capacity of hydrogels, combined with their ability to diffuse, dissolve, and release drugs, allow for precise control over the rate and timing of local release of anti-inflammatory molecules and drugs (e.g., curcumin and 7,8-dihydroxyflavone [7,8-DHF]). Curcumin, a natural antioxidant and anti-inflammatory compound, has been widely used to treat inflammatory disorders. When loaded into chitosan or hyaluronic acid hydrogel scaffolds, curcumin enables slow release, which inhibits post-SCI inflammation and improves the local microenvironment of the injured spinal cord (Luo et al., 2021; Xiao et al., 2023). Similarly, 7,8-DHF, another small molecule, exhibits neuroprotective and anti-inflammatory effects. Its encapsulation in a hydrogel promotes slow release, aiding long-term recovery. Under low-voltage stimulation, the release of 7,8-DHF stimulates dorsal root ganglion (DRG) neurite growth, Schwann cell migration, myelination, synapse formation, and enhances neuronal mitochondrial biosynthesis (Sun et al., 2022). The controlled release of anti-inflammatory drugs, antihistamines, corticosteroids such as cyclooxygenase-2 inhibitors, amino acid-based drugs, and antiviral agents can restrain the production and release of inflammatory mediators and reduce the movement of inflammatory cells, thus inhibiting inflammation (Courtine and Sofroniew, 2019).

### Conductive scaffolds and electrical stimulation

Conductive scaffolds are specialized biomaterials with intrinsic electrical conductivity, allowing them to transfer biological currents while providing a framework for cellular organization and growth (He et al., 2020). In the inflammatory microenvironment, these scaffolds have two main functions: maintaining structural integrity in the injured area and actively influencing cells and immune responses when coupled with electricity. This combination makes conductive scaffolds promising platforms for repairing tissue and modulating postinjury inflammation (Qin et al., 2023). By delivering focused electrical signals to surrounding cells, conductive scaffolds enhance metabolic activity, promote growth and flow, and accelerate repair of injured regions. Electrical input can also influence cytokine output patterns and immune cell activity, resulting in reduced inflammatory signaling, improved local conditions, and an environment more conducive to regeneration (Liu et al., 2024b).

Electrical stimulation itself affects inflammation through several mechanisms: it activates autophagy-related pathways, helps clear cellular debris and inflamed cells, and reduces overall inflammation (Li et al., 2023a). Furthermore, it optimizes the intracellular milieu by regulating calcium homeostasis, redox balance, and subsequent NSC differentiation and neurite extension (Lai et al., 2023). Conductive scaffolds allow precise, localized delivery of electrical signals, making therapy more effective and less disruptive to nearby tissue. For example, when external electrical stimulation was applied to PDA@CNT-PEDOT-grown DRG neurons, long neurites formed, Schwann cell migration from DRG spheres increased, and myelination was promoted (Reddy et al., 2019). Taken together, combining conductive scaffolds with controlled electrical stimulation is a potent approach for reconfiguring inflammatory microenvironments and advancing tissue engineering in spinal cord repair (Sun et al., 2022).

### Extracellular matrix reassembly and growth factor delivery

More recently, interdisciplinary developments at the intersection of neuroscience and biomaterials have opened new avenues for neural regeneration and repair. Biomaterials help create environments surrounding neurons to promote recovery. Nerve damage, whether caused by trauma or disease, frequently leads to the loss of neurons and axons, disrupting neural transmission and limiting functional outcomes. Conventional therapeutic methods generally fail to restore the function of damaged nerve tissue; thus, novel approaches that maintain neuronal survival and ensure nerve fibers can grow, branch, and reconnect are needed to regenerate synaptic connections. In this context, biomaterial-based systems aim to rebuild the ECM and deliver growth factors to restore structural integrity and reactivate intrinsic repair mechanisms.

Cystic cavities sealed with dense scars form after traumatic SCI, becoming a major obstacle to neural regeneration and functional recovery. The main component of these scars is the ECM, composed of numerous proteins and proteoglycans forming a supportive framework; in a normal spinal cord, cells can grow and migrate freely. Another approach is to make the ECM more permissive by modifying key inhibitory components such as chondroitin sulfate proteoglycans (Burnside and Bradbury, 2014) to promote beneficial anti-inflammatory responses. Biomaterials can induce anti-inflammatory responses in infiltrating glial cells/macrophages and ECM molecules, promoting the assembly of a fibrotic matrix instead of scar tissue. The newly arranged ECM forms an environment favorable for axonal regeneration and synaptic junction formation between endogenously derived nerve cells. New tissue formation is achieved by remodeling the ECM with biomaterials that mimic natural microenvironments, providing neuronal support for positioning and growth (Liu et al., 2020a; Sun et al., 2023b).

Hydrogels, a class of biomaterials used in regenerative medicine, have shown tremendous potential for treating SCI. Their unique properties—high biocompatibility and tunability—allow them to be designed with scaffold structures and loaded with growth factors and other bioactive molecules. Growth factors are crucial molecules regulating neuronal growth and connectivity, including nerve growth factor, brain-derived neurotrophic factor, fibroblast growth factor, and vascular endothelial growth factor, all playing vital roles in promoting neural regeneration, suppressing inflammation, and enhancing microvascular formation (Luo et al., 2021; Feng et al., 2023a; Xiao et al., 2023). Growth factors loaded into hydrogels support damaged neuron regeneration by accelerating NSC proliferation and differentiation. The rate of growth factor release and cell growth can be controlled according to each phase of injury repair. Hydrogels loaded with growth factors show great promise for modulating the SCI microenvironment (Liu et al., 2020a). By repairing tissues, promoting neural regeneration, and reducing inflammation, these treatments provide new hope for SCI therapy. However, more clinical and technical research is needed to make them more convenient and safer, offering a new approach for disease treatment.

Some studies have applied biomimetic design principles to engineer biomaterials that replicate the physical and chemical properties of the natural ECM, providing essential structural support and biochemical cues for neural repair (Feng et al., 2023b; Chu et al., 2024; Tan et al., 2024). F-SAP molecular solutions can be electrospun using hexafluoroisopropanol to form biodegradable scaffolds that mimic ECM microstructures to promote neural differentiation, neurite extension, and synaptogenesis required for regrowth (Zhao et al., 2018). Biodegradable biomaterials are advantageous because they are biocompatible and degrade slowly as cells regenerate, meaning the problem can be reversed long-term. By manipulating ECM morphology and controlling the spatiotemporal release of growth factors, these materials can help axons from damaged neurons extend to specific areas and form neural networks. In other words, beyond facilitating axon extension, biomaterials provide precise chemical and physical cues that aid new synapse formation and promote reconstruction of functional neuronal networks.

In summary, biomaterials and microenvironment engineering therapies hold tremendous potential for regulating the inflammatory microenvironment. Hydrogels, conductive scaffolds with electrical stimulation, and techniques such as biomaterial-mediated ECM reassembly and combined growth factor delivery offer new avenues for SCI regeneration. Future research should further explore the mechanisms of these biomaterials and microenvironment engineering technologies to optimize treatment strategies.

### Pharmacological modulation and molecular-targeted therapies

Neuroprotection, one of the most important neurorestorative strategies, is essential in the acute and subacute phases of SCI. During the acute phase of SCI, the aim is to minimize and/or prevent secondary medullary lesion extension, thereby decreasing apoptosis or necrosis and promoting neuronal cell and axonal survival (Huang et al., 2020). At an early stage, high-dose methylprednisolone (MP) therapy was once regarded as beneficial for neurological restoration during the acute phase of SCI (Bracken et al., 1997). Only MP has shown efficacy in clinical trials and thus has been approved for the treatment of patients with TSCI. It binds to glucocorticoid receptors to prevent the nuclear translocation of proinflammatory transcription factors, thereby inhibiting the production and release of proinflammatory signaling molecules (cytokines and chemokines) and limiting membrane lipid peroxidation (Xue and Yong, 2020; Lin et al., 2022). Experimental studies conducted on laboratory animals have revealed the beneficial effects of MP following acute SCI, particularly on white matter oligodendrocytes, when the drug is administered early (Olby et al., 2020; Metwally et al., 2022). The continuous application of MP beginning one hour after injury is associated with a significantly reduced number of pyknotic nuclei and a corresponding increase in the number of surviving astrocytes and oligodendrocytes in the ventrolateral region of the white matter, despite the presence of a considerable quantity of dead cells (Samano and Nistri, 2019).

The most prominent study supporting the use of MP was the Second National SCI Study (NASCIS II), which revealed a significant advantage in the early restoration of motor function among a subset of patients receiving MP therapy compared to those receiving a placebo (Bracken et al., 1990). Our team conducted a meta-analysis of 16 studies (1863 participants), including 3 randomized controlled trials and 13 observational studies. Based on the current evidence, compared with the control treatment, high-dose MP treatment did not help improve neurological functional recovery, but may increase the risk of adverse events in ASCI patients (Liu et al., 2019a). Therefore, recent studies have provided insufficient evidence to support the use of high-dose MP therapy for neurological restoration in patients with acute SCI. In addition, high MP doses must be administered as a pulse therapy, which may induce side effects such as sepsis, gastrointestinal bleeding, and thromboembolism (Huang et al., 2020; Xue and Yong, 2020).

Clinically, methylprednisolone (MP) may still be considered in selected cases of incomplete cervical medullary lesions, particularly for patients with cervical spondylotic myelopathy who require decompression. However, if MP is administered, strict caution is warranted, with attention to the following recommendations: (a) Adherence to the therapeutic time window is critical and should occur within 8 hours of injury. For patients receiving high-dose MP, both the infusion rate and dosage must be carefully calibrated based on accurate body weight measurements. If treatment is initiated within the first 3 hours after SCI, a bolus dose of 30 mg/kg should be infused over 15 minutes, followed by a continuous infusion of 5.6 mg/kg per hour for 23 hours. If treatment begins between 3 and 8 hours after injury, the bolus dose remains unchanged, but the subsequent continuous infusion should be reduced to 5.4 mg/kg per hour. (b) In patients whose neurological symptoms resolve prior to the completion of the regimen, MP should be discontinued immediately to minimize the risk of adverse events. (c) High-dose MP is contraindicated in patients without neurological deficits, those with penetrating or gunshot injuries to the spinal cord, those whose injuries occurred more than 8 hours prior, and in patients with gastrointestinal bleeding, diabetes, or advanced age, especially those at elevated risk of pneumonia.

Recently, novel drug delivery systems are being developed to enhance the physicochemical properties of glucocorticoids while mitigating the undesirable side effects associated with their systemic administration (Li et al., 2022b; Zhai et al., 2023). There is a pressing need for more effective delivery strategies to achieve better therapeutic efficacy against traumatic spinal cord injury (TSCI) while minimizing side effects.

## Cross-Scale Integration, Mechanistic Insights, and Translational Research Priorities

SCI leads to acute inflammation, with various cell types observed in the lesion area. This inflammatory response is primarily triggered by large cells such as macrophages and microglia, which increase the expression of genes that alter the local environment around the spinal cord near the injury site. During this time, reactive astrocytes migrate toward the center of the lesion, forming a glial scar. Although the formation of a glial scar pathologically restricts neural regeneration and acts as a barrier to both physical and biochemical axon regrowth, the shift in the polarity of reactive astrocytes during the subacute phase has neuroprotective functions (Boghdadi et al., 2020; Kisucka et al., 2021). Furthermore, SCI significantly alters the local microenvironment, primarily due to the rapid and strong responses of resident astrocytes and microglia, which can lead to extensive neuronal loss. Following injury, microglia become activated and can differentiate into either the M1 phenotype, which is pro-inflammatory, or the M2 phenotype, which is anti-inflammatory and reparative (Kisucka et al., 2021). SCI creates an inhibitory environment in which M1 predominates. Notably, the neuroprotective roles of microglia and astrocytes are positively correlated with their protective phenotypes. Evidence suggests that reactive microglia and astrocytes can switch to neuroprotective phenotypes, contributing to spontaneous motor recovery after SCI. Therefore, pharmacologically inhibiting neuroinflammation in M1 microglia, especially those generating A1-reactive astrocytes, may offer therapeutic benefits. Additionally, strategies have been developed to promote M2 macrophage polarization and recruitment to the lesion site, facilitating tissue repair and regeneration (Kong and Gao, 2017). The composition of the gut microbiota significantly affects various physiological processes and overall host health. Although the precise molecular mechanisms underlying the gut–central nervous system–immune system axis remain unclear, probiotics that modulate gut microbiota have emerged as a viable therapeutic tool for treating SCI-induced gut inflammation and restoring immune homeostasis. In animal experiments, the application of Lactobacillus probiotics to SCI mice triggered a protective immune response in the gut-associated lymphoid tissue and improved motor recovery (Kigerl et al., 2016). Research by Lu et al. (2018) revealed that a ketogenic diet alleviates oxidative stress and inflammation following SCI by activating the Nrf2 pathway and inhibiting the NF-κB pathway.

Rehabilitation and exercise are among the most effective non-invasive strategies for treating SCI. Studies have shown that various forms of rehabilitation, including treadmill training, swimming, and physical therapy, significantly support functional regeneration of the spinal cord, which is crucial for motor and sensory recovery (Li et al., 2019; Giagiozis et al., 2025; Kilgard et al., 2025). Molecular analysis of pathways involved in long-term assisted training after SCI can provide valuable insights for future rehabilitation studies (Kiss et al., 2022). High-intensity exercise has also been shown to significantly suppress the immune response following SCI (Walsh et al., 2023). However, the timing of post-injury rehabilitation is critical. Marques et al. (2018) found that compared to early treadmill training starting 7 days post-injury, later training (14–28 days post-injury) improved functional outcomes, preserved motor neurons, and increased brain-derived neurotrophic factor expression.

In recent years, significant progress has been made in understanding the effects of multicellular interactions and their molecular crosstalk on the response to SCI. A detailed understanding of glial-neuronal interactions in the lesion microenvironment can aid in the development of promising therapeutic strategies. During the acute and subacute phases, the spinal cord displays an often underappreciated plasticity. Early modulation of the inflammatory response following SCI, regulation of glial-neuronal crosstalk around the lesion center, and the time-dependent transformation of reactive microglia and astrocytes into neuroprotective phenotypes are key steps in effectively treating traumatic SCI. Additionally, addressing the disruption of gut ecology induced by SCI is an effective approach to reducing inflammatory cytokines and alleviating severe inflammation at the lesion site. Future research should focus on studying the pathology of SCI both locally and systemically (Lukacova et al., 2021).

Finally, single intervention strategies are often insufficient to effectively promote rehabilitation after SCI. Future clinical interventions may benefit from synergistic combinations of therapies. Strengthening collaboration among researchers across various fields of neuroscience to develop comprehensive treatment strategies that combine surgical and biological rehabilitation is crucial. Given the high disability rate among SCI patients, psychological interventions are also extremely important. Rehabilitation and regeneration are equally essential for these patients. In the future, AI technologies, such as brain-computer interfaces and smart devices, are expected to merge with rehabilitation technologies, potentially leading to further breakthroughs in treatment.

## Current Status of Clinical Translation and Application

Translating spatiotemporal-targeted interventions from bench research to clinical application requires coordinated efforts that combine scientific innovation, clinical proficiency, and rigorous ethical oversight to deliver tangible benefits for patients. As discussed earlier, numerous preclinical investigations, including studies involving nonhuman primates, have demonstrated both the feasibility and therapeutic potential of spatiotemporal regulation following SCI. However, no treatment has yet gained U.S. Food and Drug Administration approval for even partial neurological restoration. Nevertheless, several clinical trials have produced encouraging results and helped refine priority research directions for SCI (Fehlings et al., 2025).

We reviewed 65 clinical trials focusing on the inflammatory microenvironment in patients with SCI, utilizing data from ClinicalTrials.gov (**Additional Table 2**). Many studies concentrated on chronic inflammation and its secondary complications, such as cardiovascular issues, neuropathic pain, and depression, while assessing the safety and efficacy of various treatments. These treatments included adaptive exercise programs, functional electrostimulation (FES), medications like salsalate and statins, transcutaneous auricular vagus nerve stimulation, several stem cell-based therapies, and dietary supplements such as ketogenic or anti-inflammatory diets. Most studies employed randomized controlled trial designs to evaluate changes in levels of C-reactive protein (CRP), IL-6, mental health status, and daily activities. Sample sizes range from 8 to 256 participants, with the common goal of generating strong, evidence-based treatment recommendations for individuals with SCI in their daily lives.

**Additional Table 2:** Representative clinical program

Intervention type	Specific method	Research objective	Key data (sample size/duration)	Core indicators
Exercise intervention	12-wk adaptive exercise	Evaluate its effect on reducing inflammation (such as CRP, IL-6)	24 participants/12 wk	Changes in inflammatory biomarkers
	FES rowing exercise	Enhance pain management, reduce brain inflammation, increase hippocampal volume, and improve endogenous opioid function	13 participants/12 wk	PROMIS pain scale, MRI hippocampal volume, inflammatory biomarkers
	Aquatic physiotherapy	Improve balance, gait, and systemic inflammatory markers	50 participants/6 wk	Berg Balance Scale, levels of 20 cytokines
Pharmacological intervention	Salsalate (2g, twice daily)	Reduce fasting and postprandial lipids, glucose, and vascular inflammation-related CVD risk	18 participants/30 d	Triglycerides, glucose AUC, IL-6 levels
	Pregabalin	Prevent or alleviate central neuropathic pain and improve pain regulation mechanisms	50 participants/2-3 mon	McGill Pain Questionnaire, pain regulation ability (VAS score)
	Statins (Rosuvastatin)	Reduce bone loss and inflammation, lower endocrine-metabolic disease risk	8 participants/12 mon	Bone density, hsCRP, IL-6, TNF-α levels
Neuromodulation	taVNS (1 h / 30 d)	Evaluate its anti-inflammatory, antidepressant, and autonomic function effects	44 participants/30 d	Inflammatory biomarkers (TNF-α, IL-6), PHQ-9 depression scale, heart rate variability
	Non-invasive vagus nerve stimulation	Reduce sympathetic nerve activity and chronic inflammation	0 participants (withdrawn)	Heart rate variability, cytokine levels
Stem cell therapy	Neural cells (Neuro-Cells)	Assess safety of intrathecal injection in chronic traumatic SCI patients	10 participants/3 mon	ISNCSCI checklist, incidence of adverse events
	MSC	Verify safety of adipose-derived MSC intrathecal injection	10 participants/96 wk	Adverse events, ASIA score
Dietary intervention	Ketogenic diet	Improve motor-sensory function, blood glucose, and functional independence	60 participants/5 wk	ASIA motor score, blood glucose level, SCIM score
	Anti-inflammatory diet	Improve adherence, reduce neuropathic pain and depression	11 participants/1 mon	Dietary adherence rate, NPQ pain scale, CES-D depression scale
	Intermittent fasting (16 h/d)	Reduce inflammation and depressive symptoms	32 participants/8 wk	Inflammatory biomarkers, PHQ-9 depression scale
Surgical intervention	Duroplasty	Evaluate its effect on functional recovery in severe cervical SCI patients	222-260 participants/1 yr	ASIA motor score, WISCI II walking index, quality of life (SF-36)

AUC: Area under the curve; ASIA: American Spinal Injury Association; CES-D: Center for Epidemiologic Studies Depression Scale; CRP: C-reactive protein; CVD: cardiovascular disease; FES: functional electrical stimulation; hsCRP: high-sensitivity C-reactive protein; IL-6: interleukin-6; ISNCSCI: International Standards For Neurological Classification of Spinal Cord Injury; MSC: mesenchymal stem cells; MRI: magnetic resonance imaging; NPQ: Neuropathic Pain Questionnaire; PHQ-9: Patient Health Questionnaire-9; PROMIS: Patient-Reported Outcomes Measurement Information System; SCIM: Spinal Cord Independence Measure; SF-36: 36-item Short Form Health Survey; taVNS: transcutaneous auricular vagus nerve stimulation; TNF-α: tumor necrosis factor alpha; VAS: Visual Analog Scale; WISCIII: Walking Index for Spinal Cord Injury II.

Among the interventions assessed, exercise has been studied most extensively. Both immediate and delayed 12-week adaptive exercise programs have decreased circulating pro-inflammatory indicators such as CRP and IL-6, indicating that physical activity has anti-inflammatory effects in patients with SCI treated with FES. This includes patients with paraplegia, who reported less pain and increased hippocampal volume after more than three months of treatment, which also reduced cell damage. Engaging larger muscle groups may thus improve systemic immune defense and protect sensitive tissues. Similarly, a patient who underwent a 6-week aquatic therapy regimen for subacute, incomplete SCI (T1–L5, ASIA C/D) showed improved balance (Berg Scale) and gait (10-meter walk test), along with the downregulation of 20 inflammatory mediators after swimming. This indicates the benefits of reduced weight bearing and safer mobility in water.

In current clinical protocols targeting inflammation, therapeutic benefits are typically achieved through the downregulation of cytokines such as IL-6 and TNF-α. Moreover, exercise, dietary regulation, and neuromodulation are selected to balance safety and compliance. Subacute-phase interventions tend to promote recovery, such as aquatic therapy, whereas chronic-phase interventions address symptoms like neuropathic pain or metabolic complications. While the results are promising, limitations remain. Most studies involve small cohorts (usually 10–50 participants), which reduces statistical power. Follow-up periods often end before 12 weeks, leaving long-term effects unclear. Some treatments, such as FES and robotic exoskeletons, require specialized equipment, limiting accessibility. Future studies should prioritize large-scale, multicenter randomized controlled trials to explore the synergistic effects of combined approaches—such as exercise and diet—and further investigate dietary modifications in managing metabolic disorders. Refining treatment assessments using biomarker panels, including inflammatory profiles and gut microbiota composition, is also essential.

## Limitations

Although numerous methodologies have been developed to assess the spatiotemporal changes in inflammatory microenvironments, very few of these are experimental. As a result, some limitations may exist regarding the precise magnitude of the pathophysiological changes reviewed. Furthermore, excluding non-English publications has narrowed our scope.

## Discussion

SCI remains one of the most formidable neurological disorders, characterized by limited restorative capacity and complex pathophysiological mechanisms that hinder functional recovery. Although recent decades have seen advances in understanding the progression and repair of SCI, translating these findings into effective clinical therapies remains challenging. This review highlights current insights into the spatiotemporal changes in the inflammatory microenvironment in SCI and examines how timely, location-specific management can affect neural regeneration. We place this framework within a broader research context to present new ideas, practical applications, and directions for future investigation.

The field of neuroinflammation has been shaped by decades of foundational discoveries. For instance, while microglia were once considered solely pro-inflammatory cells, they can also adopt regenerative phenotypes during the subacute phase to clear debris, release neurotrophic factors, and support axonal sprouting (Lu et al., 2025a). These observations suggest a therapeutic benefit of selective immunomodulation. High-resolution imaging and molecular profiling have refined our understanding of the spatiotemporal characteristics of SCI. Single-cell RNA sequencing has revealed significant heterogeneity among neural progenitors, astrocytes, and immune cell populations across different spinal cord regions post-injury (Luo et al., 2021). More recently, spatial transcriptomics has enabled *in situ* mapping of gene expression patterns, revealing localized cellular responses confined to specific microenvironments (Rodgers et al., 2022). Collectively, these findings challenge the notion of a universal therapy and underscore the need for targeted, precise treatments. Despite considerable progress, several key problems persist. Traditional anti-inflammatory approaches, such as corticosteroids like MP, have proven largely ineffective for long-term improvement due to their non-discriminatory suppression of both harmful and beneficial immune responses (Hurlbert et al., 2013). Similarly, targeted approaches, such as antibody depletion strategies designed to eliminate specific immune subsets, can inadvertently disrupt homeostatic pathways or trigger compensatory responses that worsen injury (Gao et al., 2024). Moreover, no unified framework exists to define “good” *versus* “bad” immune responses across the injury continuum, from the acute (0–72 hours) to chronic (weeks to months) phases.

This review adopts a spatiotemporal framework to link cellular heterogeneity to evolving functional states along the post-injury timeline. Unlike earlier reviews that focused narrowly on individual cell types or static features of the injury microenvironment (Fan et al., 2022; Hu et al., 2023), this review offers a more integrated, microenvironment-dependent model. In this perspective, immune cell functions cannot be uniformly categorized as pro- or anti-regenerative; rather, their roles depend on anatomical location and temporal stage. For instance, astrocytes at the lesion core often contribute to glial scar formation, whereas those in the surrounding gray matter may support axonal survival through the release of trophic factors—differences that are rarely emphasized in previous studies.

Another major innovative element of this review is its focus on the synergistic potential of new technologies within our STDIM framework. We combined spatial transcriptomics with organoid models and employed AI-driven predictions to overcome the limitations of traditional *in vitro* and *in vivo* models. Spatial transcriptomics can generate region-specific gene expression patterns, while AI algorithms can predict the outcomes of manipulating certain cell types at specific time points. Together, these complementary tools may lead to highly tailored, precision therapies (Xu et al., 2023; Han et al., 2025).

In this review, we revisit the traditional separation between the suppression of inflammation and the promotion of regeneration. We argue that the optimal approach is to modulate rather than eliminate immune activity, aligning therapy with natural healing processes. In this context, divergent results among studies reporting pro- or anti-inflammatory interventions may be reconciled (Kigerl et al., 2016).

The contribution of this review lies in providing a unified framework for interpreting immune-neural interactions in SCI. Focusing on the anatomical and temporal aspects of inflammation offers an opportunity to integrate conflicting results across studies. Moreover, the concepts proposed here for SCI may extend to other neuroinflammatory disorders, such as traumatic brain injury and stroke, where similar spatial and temporal immune effects have been documented (Ziebell and Morganti-Kossmann, 2010). This review also enhances the understanding of how immune function affects CNS injury and repair.

Currently, in clinical settings, treatment for SCI is primarily supportive, focusing on decompressive surgery and rehabilitation, with no proven therapies that facilitate true neural regeneration (Fehlings et al., 2021). Rather than employing broad immunosuppression, a more precise spatiotemporal modulation that promotes pathogen clearance and tissue reconstruction while preventing cytokine storms or excessive scarring may be preferable. Strategies have been explored that involve the acute, localized delivery of anti-inflammatory agents to the lesion core, followed by the subacute delivery of pro-regenerative agents to the perilesional tissues. This approach emphasizes precision beyond current personalized medical treatments. Furthermore, the STDIM framework provides insights for reinterpreting the “failures” of earlier trials, such as systemic anti-TNF therapy, which likely did not succeed due to poor timing or targeting (Bai et al., 2025).

Based on these results, several future priorities can be identified. First, longitudinal spatial mapping of human SCI tissue should be conducted to determine whether the spatiotemporal patterns observed in preclinical models are genuine. Rodent studies have revealed critical but species-specific differences in immune cell function, making human datasets indispensable. Emerging tools, such as minimally invasive biopsy approaches combined with single-cell spatial transcriptomics, could make this analysis feasible, although ethical challenges and tissue accessibility must be addressed.

Second, advancements in targeted delivery technologies will facilitate the clinical translation of the STDIM framework. For example, nanoparticles designed to aggregate in specific spinal cord regions or engineered to release therapeutic payloads in response to injury-induced signals, such as changes in pH or cytokine distribution, could provide precise yet systemically nontoxic modulation (Li et al., 2025). Additionally, cell type-specific gene editing via viral vectors could produce engineered astrocytes or microglia for long-term repair in a space/time-dependent manner (Barclay et al., 2024; Zhou et al., 2025).

Third, a multidimensional preclinical platform integrating organoid systems, live injury models, and computer simulations should be established (Means et al., 2025). This platform could accurately predict therapeutic outcomes. Human induced pluripotent stem cell-derived organoids allow for the replication of individual-specific immune-neural interactions, while AI-driven in silico approaches can forecast the effects of spatially and temporally targeted interventions on long-term recovery (Meng et al., 2023; Luo et al., 2025). Combining these techniques could reduce reliance on animal studies, shorten timeframes, and accelerate clinical translation.

Finally, it is essential to build robust interdisciplinary networks linking immunology, neuroscience, bioengineering, and clinical disciplines. Historically, SCI research has been fragmented, with researchers in different fields focusing narrowly on inflammation or regeneration. Promoting interdisciplinary collaboration can foster an integrated approach that addresses both simultaneously (Picetti et al., 2024). International coalitions, such as the Spinal Research Network (https://spinal-research.org/home/our-research/the-research-network/), provide valuable platforms for data sharing, protocol harmonization, and reproducibility between institutions, ultimately accelerating the adoption of evidence-based interventions (Muller et al., 2022).

## Conclusions

The STDIM plays a critical role in the progression and repair of SCI. The pathophysiological mechanisms of SCI involve diverse cellular processes, inflammatory responses, and scar formation, necessitating a multifaceted, multitargeted approach for effective treatment. This review highlights that spatiotemporal-targeted immunopriming is a promising treatment for SCI. It reconciles divergent findings from various studies, underscores technological advancements, and outlines a pathway for translating these therapies into practice for treating patients with SCI. This work enhances our understanding of how to harness the immune system to stimulate nerve repair.

Although many obstacles remain, pursuing precise, context-dependent regulation is crucial for overcoming these challenges. Elucidating the correct spatiotemporal characteristics of the inflammatory microenvironment presents an opportunity to develop more precise, stage-specific interventions that can suppress harmful responses while preserving beneficial immune functions. These interventions are expected to enhance regeneration, mitigate secondary injury, and ultimately improve neurological recovery in patients with SCI.

## Additional files:

***Additional Table 1:*** *Summary of spatiotemporal disruption of the inflammatory microenvironment (STDIM).*

***Additional Table 2:*** *Representative clinical program.*

## Data Availability

*All relevant data are within the paper and its Additional files.*
